# Perceived cultural continuity, self-schemas, self-esteem, and psychological distress among members of ethnic groups with endangered scripts in Southwest China

**DOI:** 10.3389/fpsyg.2026.1768184

**Published:** 2026-02-11

**Authors:** Xian Zhang, Zichan Li

**Affiliations:** 1 Academic Affairs Office, Hunan First Normal University, Changsha, China; 2 Department of Chinese Music, Singapore Raffles Music College, Singapore, Singapore

**Keywords:** southwestern ethnic minorities, perceived cultural continuity, psychological distress, self-esteem, schema construction

## Abstract

**Introduction:**

Ethnic minorities whose languages and traditional scripts are being eroded in everyday use and education may experience weakened continuity of collective memory, identity, and values, thereby heightening their vulnerability to psychological distress. This study examines how perceived cultural continuity relates to psychological distress among ethnic minorities with endangered scripts in Southwest China and whether self-schemas and self-esteem serve as key psychological mechanisms in this association.

**Methods:**

This study conducted a community-based paper-and-pencil survey in March–April 2025 among adult members of four ethnic minority groups in Southwest China whose traditional scripts are endangered (Tibetan, Yi, Dai, and Naxi). Using convenience and snowball sampling, 500 questionnaires were distributed and 458 valid responses were obtained. Structural equation modeling with maximum likelihood estimation and bootstrapping in AMOS 23 was used to test the hypothesized relationships and mediation effects.

**Results:**

The study found that perceived cultural continuity was positively associated with self-schema and self-esteem, which in turn were negatively associated with psychological distress. Specifically, self-schemas and self-esteem both linked higher perceived cultural continuity to lower psychological distress, and a significant indirect effect confirmed that self-schemas and self-esteem jointly mediated the association between perceived cultural continuity and psychological distress.

**Discussion:**

The findings indicate that stronger perceived cultural continuity is associated with more coherent self-schemas, higher self-esteem, and lower psychological distress, suggesting that the preservation of cultural continuity can be internalized into a more stable and positive sense of self. These results underscore the importance of protecting endangered scripts and cultural practices not only as heritage resources but also as psychological assets that may buffer distress in minority communities.

## 1 Introduction

A substantial body of public and mental health research documents persistent disparities in mental health outcomes between racial/ethnic minority groups and majority populations (Cho and Hamler, 2025). Prior studies suggest that ethnic minority communities may face greater mental health–related stigma, poorer access to mental health care, and a higher risk of psychological distress (Lu et al., 2021; Eylem et al., 2020; El-Refaay et al., 2025). During the COVID-19 pandemic, the mental health of Black, Hispanic, and Asian adults in the United States deteriorated more markedly than that of White adults, with significant increases in depression and anxiety reported among these groups (Thomeer et al., 2023). Evidence from US university students similarly indicates that, compared with White peers, racial/ethnic minority students report higher levels of anxiety and depression, alongside greater unmet mental health needs (Lipson et al., 2022). Population-based surveillance data in the United States further show that, between 2018 and 2023, suicide rates increased among American Indian and Alaska Native, Black, and Hispanic populations, whereas the suicide rate among non-Hispanic White populations decreased (Stone, 2025). Taken together, these findings underscore an urgent need for inclusive, culturally grounded approaches that help vulnerable communities build psychological resources and resilience, rather than relying solely on deficit-oriented models of mental health.

Within this broader agenda, growing attention has been directed to the role of cultural and identity-related assets in supporting well-being. Perceived cultural continuity refers to an individual’s subjective belief that their group’s cultural identity remains stable over time and is transmitted from one generation to the next. The construct originates in research on identity continuity, which suggests that people are more likely to maintain psychological coherence when they perceive their social groups as having a consistent history, shared traditions, and enduring collective narratives (Sani et al., 2007). Building on this foundation, recent scholarship conceptualizes perceived cultural continuity as the perceived durability of core cultural elements, including language, rituals, values, and collective historical narratives (González et al., 2022; Cobb et al., 2025). Empirical research among Indigenous and other minority populations indicates that higher perceived cultural continuity is associated with fewer depressive symptoms, greater life satisfaction, and stronger psychological well-being (Firestone et al., 2024; Masotti et al., 2023). Qualitative evidence further suggests that sustaining cultural connectedness contributes to identity stability, social integration, and emotional health (Murrup-Stewart et al., 2021). Cross-cultural work likewise links continuity of cultural identity with greater resilience and adaptive capacity in the context of social change or marginalization, and with improved psychological well-being via enhanced identity coherence (Cruwys et al., 2021). These findings position perceived cultural continuity as a promising strength-based psychological resource that may be particularly relevant for communities facing cultural disruption.

The theory of self-continuity posits that individuals’ self-awareness relies on a coherent narrative that connects past, present, and future experiences (Jiang et al., 2020). When cultural continuity is perceived as weak or disrupted, self-narratives may become fragmented, undermining identity stability and increasing vulnerability to psychological distress (Kirmayer et al., 2014). Social identity theory further suggests that identification with social groups provides predictability, meaning, and psychological security (Turner and Oakes, 1986; Stets and Burke, 2000). Consistent with these perspectives, qualitative evidence from Indigenous communities in North America suggests that cultural continuity—reflected in intergenerational knowledge transmission, cultural connectedness, and the maintenance of traditional practices—plays an important role in supporting well-being and buffering against psychological distress (Auger, 2016). Research in Indigenous and cross-cultural psychology also indicates that disruptions to cultural or collective identity are associated with heightened feelings of alienation, emotional distress, and reduced well-being (Durie, 2004; Brougham and Haar, 2013). Conceptually, perceived cultural continuity may buffer psychological distress by sustaining identity coherence and reinforcing a sense of belonging.

Following evidence that cultural continuity supports well-being, attention turns to the proximal psychological mechanisms through which this resource may operate. Self-schemas are cognitive structures that organize and guide the processing of self-relevant information, shaping how individuals interpret experiences in specific (including identity-relevant) domains (Evans et al., 2005; Markus et al., 1982). Self-esteem refers to the evaluative component of the self-concept and reflects an individual’s overall sense of self-worth (Swann et al., 2007). A substantial body of research links these constructs to mental health outcomes; for example, stable and coherent self-representations and higher global self-esteem are associated with better stress regulation and a lower risk of depressive and anxiety symptoms (Nasso et al., 2020; Hilbert et al., 2019). Evidence from Indigenous and other minority populations further suggests that cultural connectedness is related to more positive self-perceptions and better well-being (Holt et al., 2014; Verkuyten, 2003). In this light, self-schemas and self-esteem can be understood as internal psychological resources that translate culturally grounded continuity into resilience-related outcomes, including lower psychological distress.

In southwestern China, the maintenance and transmission of minority languages and cultural practices have been profoundly shaped by a specific sociopolitical and historical context. The Constitution of the People’s Republic of China and the Law on Regional National Autonomy formally guarantee ethnic minorities the right to use and develop their own languages and scripts (People’s Republic of China, 1984), while simultaneously promoting Mandarin Chinese as the national common language (Lundberg, 2009; Zhou and Lundberg, 2009). Within this legal framework, bilingual education has long been adopted as a primary policy instrument intended to balance minority language preservation with national language acquisition (Wang et al., 2024). However, empirical research indicates that, in recent decades, the implementation of bilingual education policies has increasingly prioritized Mandarin proficiency, reflecting broader national objectives related to educational standardization, social mobility, and economic integration (Xu and Liu, 2023; Zhang et al., 2022). National education planning documents emphasize early and sustained Mandarin instruction, often through standardized curricula that limit the functional use of minority languages in formal schooling (Ministry of Education of the People’s Republic of China, 2010). These educational dynamics intersect with wider processes of social modernization—including urbanization, internal migration, and the expansion of national media—which collectively reshape everyday language practices and reduce opportunities for intergenerational transmission of minority languages and cultural knowledge (Lin and Jackson, 2021; Yan et al., 2024). As a result, despite formal legal protections, the continuity of minority languages and cultural heritage in southwestern China has become increasingly fragile.

Empirical studies document this fragility across multiple minority groups in the region. For example, the Naxi language and its traditional Dongba script have been identified as increasingly endangered, with declining everyday use among younger speakers and marked disruptions in intergenerational transmission (Huang, 2025). Broader survey evidence similarly indicates that the languages of Yi communities are experiencing reduced vitality, as younger generations increasingly rely on Mandarin Chinese in both educational and daily contexts (Yao et al., 2023). These trends are consistent with global patterns of language endangerment, whereby sociopolitical and economic pressures contribute to the rapid erosion of linguistic diversity (Bromham et al., 2022). Although research in linguistics and ethnic studies has extensively examined language vitality, script transmission, and cultural preservation, far less attention has been paid to the psychological implications of perceived cultural disruption. In particular, public health and psychological research rarely integrates minority language endangerment with perceived cultural continuity and mental health outcomes within a unified empirical framework. Consequently, little is known about how structural pressures on cultural transmission are internalized as subjective perceptions of continuity, or how such perceptions may function as psychological resources that buffer distress among minority populations in this region.

Building on the theoretical and empirical literature reviewed above, the present study aims to examine the relationship between perceived cultural continuity and psychological distress among minority populations in southwestern China, with a particular focus on Tibetan, Yi, Dai, and Naxi communities. These groups were selected because each possesses a distinctive language or script that is central to cultural identity and currently faces varying degrees of endangerment. In addition, their differing population sizes and geographic distributions, ranging from the Tibetan population of over 7 million on the Qinghai-Tibet Plateau to the smaller Naxi population of approximately 11,000 in Lijiang and Liangshan, provide demographic and cultural representativeness. Specifically, we test whether self-schemas and self-esteem serve as key psychological mechanisms linking perceived cultural continuity to psychological distress. By integrating perspectives from cultural psychology, social identity theory, and self-concept research, this study aims to advance understanding of how cultural resource decline and perceived disruptions in cultural continuity relate to psychological well-being, and to identify potential targets for inclusive, culturally grounded positive psychology interventions.

This research makes three primary contributions. First, it extends the literature on perceived cultural continuity, which has largely focused on North American Indigenous populations, to a Chinese context, thereby testing the cross-cultural generalizability of previous findings. Second, it bridges the gap between cultural and psychological research by examining potential intrapersonal mechanisms, such as self-schemas and self-esteem, that may mediate the effects of cultural continuity on mental health. Finally, the study has practical implications for the design of interventions and policies aimed at promoting psychological well-being among minority populations, suggesting that initiatives supporting cultural continuity and identity maintenance may help protect against distress and strengthen self-concept stability.

The paper is organized as follows. Section 2 presents the hypotheses and conceptual framework. Section 3 describes the methods employed for data collection and analysis. Section 4 reports the results of the data analysis and evaluates the proposed hypotheses. Section 5 offers a discussion, highlighting theoretical contributions, practical implications, and limitations, and provides suggestions for future research. Finally, Section 6 concludes the paper.

## 2 Literature review and hypothesis development

### 2.1 Self-continuity theory

Self-continuity theory posits that individuals maintain psychological stability and well-being by perceiving a coherent connection between their past, present, and anticipated future (Sedikides et al., 2023). Disruptions in this perceived continuity can undermine the construction of a stable personal narrative, increasing susceptibility to stress and emotional difficulties (Chandler et al., 2003). A sense of temporal coherence is therefore essential for interpreting life events, sustaining purpose, and preserving personal meaning. Threats to self-continuity may result in identity confusion, diminished autonomy, and heightened emotional instability.

While initially developed to explain personal identity development, self-continuity theory has been extended to collective and cultural domains. At the group level, continuity, as reflected in shared historical narratives, cultural traditions and collective identity, can enhance individuals’ feelings of stability, belonging, and psychological security (Sani et al., 2008). For members of minority or culturally distinct groups, perceived cultural continuity functions as a psychological resource, anchoring the self within a stable cultural framework and supporting the integration of personal and collective narratives (Ji et al., 2019).

Perceived cultural continuity can thus be understood as a collective form of self-continuity. When cultural values, traditions, and historical narratives are perceived as enduring, individuals are more likely to experience coherence in self-concept, resilience, and well-being (Ji et al., 2022). Conversely, perceptions of cultural disruption may compromise identity coherence and elevate vulnerability to psychological distress. In this way, self-continuity theory provides a conceptual foundation for understanding how cultural continuity contributes to mental health by supporting coherent self-structures and buffering against psychological challenges.

### 2.2 Social identity theory

Social identity theory offers an important perspective for understanding how individuals derive psychological resources from their membership in social groups (Hogg, 2016). The theory proposes that people define part of their self-concept through group affiliations and through the internalization of group norms, values, and meanings (Brown, 2000; Stets and Burke, 2000). When individuals perceive a group as meaningful and positively valued, they are more likely to experience a stronger sense of belonging and coherence in their self-understanding.

A substantial body of research shows that social identification contributes to psychological well-being (Schmid and Muldoon, 2015; Albarello et al., 2021). Individuals who feel closely connected to valued social groups tend to report lower levels of psychological distress (Jackman et al., 2023), greater resilience in coping with stress (Ntontis et al., 2023), and higher perceptions of personal meaning (McNamara et al., 2021). These benefits appear to stem from several sources, including shared group norms that guide behavior, access to social support, and a sense of continuity that helps individuals situate their personal experiences within a larger collective narrative (Klik et al., 2025; Häusser et al., 2023).

Within this theoretical framework, perceived cultural continuity can be understood as a key contributor to social identity. When individuals believe that their cultural group has preserved stable traditions, values, and historical narratives over time, they are more likely to experience a coherent sense of group belonging (Amiot et al., 2015; Weststrate et al., 2024). This sense of continuity reinforces their understanding of who they are in relation to the group and provides psychological security that supports mental health. Therefore, social identity theory offers a conceptual foundation for examining the influence of perceived cultural continuity on individual well-being.

### 2.3 Hypothesis development

#### 2.3.1 Perceived cultural continuity, self-schemas, and self-esteem

Although direct empirical evidence specifically linking perceived cultural continuity to self-schemas is limited, research in cultural, social, and identity psychology provides theoretical and empirical support for this association. A growing body of work on ethnic and cultural identity suggests that when individuals perceive their cultural group as coherent, stable, and enduring, they are more likely to experience clarity in self-related cognitive processes. For example, studies on cultural identity clarity demonstrate that individuals with a well-defined sense of cultural heritage and membership tend to show higher self-concept clarity and better psychological functioning, including self-esteem and subjective well-being (Chen et al., 2024; Rahim et al., 2021). These findings imply that cultural frameworks that are perceived as meaningful and stable contribute to more organized and consistent self-structures, which are key components of self-schemas.

Further supporting this notion, research on cultural connectedness and identity engagement among Indigenous and minority populations reveals positive associations between involvement in cultural practices and various aspects of psychological functioning (Doery et al., 2023; Misra et al., 2021). Empirical evidence indicates that engagement with cultural traditions, cultural identity practices, and spiritually meaningful activities is associated with stronger self-awareness, greater confidence in one’s abilities, and higher life satisfaction (Ironside et al., 2020; Gray and Cote, 2019; Ullrich, 2019; Fuchs and Fuchs, 2020). Although these studies do not directly measure perceived cultural continuity, they illustrate how culturally embedded experiences support the organization of self-related beliefs and evaluations. On this basis, the following hypotheses are advanced:

*Hypothesis 1* (H1): Perceived cultural continuity is positively associated with self-schemas.

Similarly, research on cultural identity integration and engagement offers insights into the relationship between perceived cultural continuity and self-esteem. Individuals who perceive their cultural heritage as coherent, meaningful, and integrated tend to develop stable and positive self-evaluations. For instance, a study of Chinese vocational pathway university students found that bicultural identity integration significantly predicted higher self-esteem, academic resilience, and school belonging (Chen et al., 2022). These findings suggest that perceiving one’s cultural background as integrated and coherent can strengthen identity security and foster positive self-worth.

Additional research indicates that individuals who hold a clearer and more positive understanding of their cultural background tend to report higher self-worth and better psychological adjustment (Gummadam et al., 2016; Kapke et al., 2017). These findings suggest that a stable and coherent sense of cultural heritage may reinforce identity security, thereby promoting higher self-esteem. Moreover, studies on cultural socialization and cultural engagement further highlight the psychological benefits associated with cultural continuity (Viola et al., 2024). For example, research with Chinese Mulao adolescents has shown that cultural socialization and engagement in ethnic traditions are positively associated with stronger ethnic identity and higher self-esteem (Kuang and Nishikawa, 2021). In multicultural contexts, greater cultural identity integration has also been linked to higher levels of self-esteem (Schotte et al., 2018; Morcom, 2017). Taken together, these findings indicate that when individuals perceive their cultural heritage as continuous, coherent, and enduring, they are more likely to develop positive self-evaluations and a stronger sense of self-worth. On this basis, the following hypotheses are advanced:

*Hypothesis 2* (H2): Perceived cultural continuity is positively associated with self-esteem.

Previous research indicates that clear and positively valanced self-schemas help individuals maintain cognitive consistency when processing information and making decisions, thereby enhancing self-concept clarity and promoting psychological well-being (Cherry et al., 2020). Specifically, empirical studies across diverse populations have consistently found that greater self-concept clarity, is linked to stronger self-esteem (Kawamoto, 2020; Stenhaug and Solem, 2024), suggesting that a well-organized cognitive structure of the self provides a foundation for positive self-evaluation. Furthermore, studies show that stable self-representations help individuals preserve positive self-evaluations when facing life challenges, supporting both emotional stability and adaptive functioning (Calheiros et al., 2020). These findings collectively indicate that self-schemas serve as a key cognitive resource that reinforces self-esteem. On this basis, the following hypotheses are advanced:

*Hypothesis 3* (H3): Self-schemas are positively associated with self-esteem.

#### 2.3.2 Self-schemas, self-esteem, and psychological distress

Accumulating evidence suggests that the structure and valence of individuals’ self-schemas are closely related to their psychological well-being. Studies on self-concept clarity, which reflects the coherence of one’s self-schemas, have shown that individuals with higher levels of self-concept clarity tend to report fewer symptoms of depression and anxiety (O’Byrne et al., 2021; Hanley and Garland, 2017). Longitudinal research also demonstrates that low self-concept clarity predicts increases in perceived stress and depressive symptoms over time, suggesting that unclear or incoherent self-schemas can contribute to the development and maintenance of psychological distress (Hertel et al., 2024). Complementary work indicates that self-related constructs, including self-concept clarity, are uniquely and negatively associated with both depressive and anxiety symptoms in non-clinical samples, highlighting the importance of coherent self-representations for emotional health (Singhal et al., 2025; He et al., 2021). These findings collectively suggest that maladaptive or poorly integrated self-schemas may heighten vulnerability to psychological distress, whereas clearer and more stable self-schemas may confer resilience. On this basis, the following hypotheses are advanced:

*Hypothesis 4* (H4): Self-schemas are negatively associated with psychological distress.

Extensive research has established self-esteem as a key psychological resource that contributes to lower levels of psychological distress (Duran et al., 2021; Liu et al., 2021). Individuals with higher self-esteem are generally better able to manage stress, maintain emotional stability, and exhibit adaptive coping behaviors, whereas low self-esteem increases vulnerability to negative emotional outcomes. Self-esteem has been conceptualized not only as a global evaluative dimension of the self but also as a protective factor that buffers the negative effects of stressors on mental health across diverse populations (Dentale et al., 2020). Consistent with this, recent evidence from a study of infertile Chinese women demonstrates that self-esteem not only negatively predicts depression and anxiety but also mediates and moderates the impact of infertility-related stress on psychological distress (Cui et al., 2021). Longitudinal and prospective studies further indicate that low self-esteem is associated with increases in depressive symptoms and anxiety over time, while higher self-esteem predicts better emotional adjustment and resilience (Choi et al., 2019; Rossi et al., 2020). Moreover, research suggests that self-esteem can attenuate the adverse effects of situational stressors, such as social rejection or health-related challenges, on psychological well-being (Rojo López et al., 2021; Freedman and Dainer-Best, 2024). Collectively, these findings underscore self-esteem’s protective role against psychological distress and its function as a stable psychological resource that supports emotional regulation and adaptive coping. On this basis, the following hypothesis is advanced:

*Hypothesis 5* (H5): Self-esteem is negatively associated with psychological distress.

#### 2.3.3 Mediation effects

Prior studies have shown that culturally grounded identity processes play an important role in shaping self-related cognitive structures (Szabo and Ward, 2015; Zhu and Lin, 2022). Cultural identity clarity and cultural connectedness are consistently associated with more coherent self-concepts, greater self-concept clarity, and more stable self-evaluations, which are core features of adaptive self-schemas (Rahim et al., 2021; Chen et al., 2024). These findings suggest that perceiving one’s cultural background as continuous and stable may contribute to the formation of more organized and positively valanced self-schemas by offering a consistent symbolic framework for self-definition.

In turn, a substantial body of longitudinal and meta-analytic research indicates that self-schemas and self-esteem are closely linked to psychological distress. Negative or fragmented self-schemas are associated with elevated levels of depressive and anxiety symptoms (Evans et al., 2005), whereas positive self-schemas and higher self-esteem function as psychological resources that buffer against distress (O’Byrne et al., 2021; Liu et al., 2021). Moreover, self-esteem has been shown to partially mediate the association between self-related cognitive structures and mental health outcomes, highlighting its role as a proximal determinant of emotional well-being (Duy and Yıldız, 2019; Fu et al., 2021). Taken together, these lines of evidence suggest a theoretically plausible indirect pathway in which perceived cultural continuity contributes to more adaptive self-schemas, which in turn foster higher self-esteem and lower psychological distress. On this basis, the following hypothesis is advanced:

*Hypothesis 6* (H6): Perceived cultural continuity is negatively associated with psychological distress through the mediating roles of self-schemas and self-esteem.

**Figure 1:**
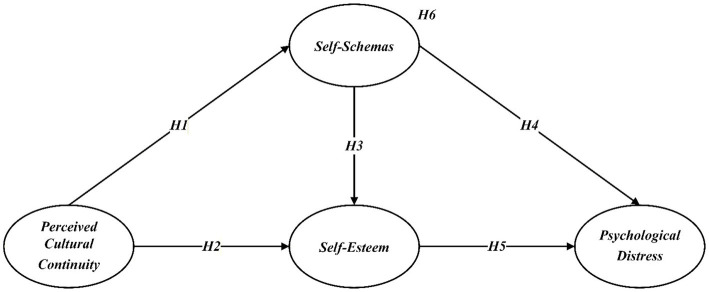
Hypothesis model.

Figure 1 provides an overview of all hypotheses.

## 3 Methodology

### 3.1 Participants and procedures

The participants in this study were adult members of four ethnic minority groups in Southwest China whose traditional scripts are endangered, including the Tibetan, Yi, Dai, and Naxi ethnic groups. From March to April 2025, researchers conducted a paper-based questionnaire survey in communities located in the main settlement areas of these groups in Southwest China. Due to practical constraints related to access, geographic dispersion, and the absence of comprehensive population registers for these minority communities, convenience sampling and snowball sampling were employed. Specifically, the researchers first contacted local community organizations and key informants to invite eligible members of the target ethnic groups to complete the questionnaire. These initial participants were then asked to refer other members of their ethnic group who met the inclusion criteria. All participants were informed of the purpose of the survey and participated voluntarily before filling out the questionnaire.

A total of 500 questionnaires were distributed and 482 were returned. After excluding invalid questionnaires (e.g., those with substantial missing data or clear response patterns), 458 valid questionnaires were retained for analysis, yielding an effective response rate of 91.6%.

The demographic characteristics of the participants are presented in Table 1. Overall, 43.9% of the respondents were male and 56.1% were female. In terms of age, 18–29, 30–39, 40–49, and 50–60 years accounted for 23.4, 28.6, 29.7, and 18.3% of the sample, respectively. Regarding ethnicity, 34.5% were Tibetan, 31.9% Yi, 22.1% Dai, and 11.6% Naxi. Most participants had a three-year college (associate) degree (59.8%), followed by undergraduate (19.9%) and postgraduate or above (7.2%). For family economic status, 55.2% reported being about average, with 35.1% reporting lower-than-average and 9.6% higher-than-average economic status.

**Table 1:** Demographic characteristics.

Profiles	Options	%
Gender	Male	43.9
	Female	56.1
Age	18–29	23.4
	30–39	28.6
	40–49	29.7
	50–60	18.3
Ethnicity	Tibetan	34.5
	Yi	31.9
	Dai	22.1
	Naxi	11.6
Education level	Junior high school or below	2.0
	Senior high school or technical secondary school	11.1
	Three-year college (associate degree)	59.8
	Undergraduate (bachelor’s degree)	1.9
	Postgraduate or above	7.2
Family’s economic status	Much lower than average	12.0
	Slightly lower than average	23.1
	About average	55.2
	Slightly higher than average	7.0
	Much higher than average	2.6

### 3.2 Instruments

The questionnaire consisted of five sections. The first section asked respondents to report their demographic information, including gender, age, ethnicity, education level, and family economic status. The second section contained six items assessing respondents’ perceived cultural continuity, such as “Our ethnic group has passed on its traditions across different generations” (Sani et al., 2007).

The third section included eight items from the scale revised by Campbell et al. (1996) to measure self-schemas, with sample items such as “In general, I have a clear sense of who I am and what I am.” Although this scale was originally developed to assess self-concept clarity, it is used in the present study to capture the structural clarity and coherence of self-related cognitive representations, which constitute a core structural dimension of self-schemas. In this sense, self-schemas are conceptualized as the degree to which self-beliefs are clearly defined, internally consistent, and stable, rather than the specific content of identity or role-based self-definitions. This construct is conceptually related to, but distinct from, identity clarity or self-concept consistency, as it focuses on the organization and coherence of self-knowledge rather than the clarity of specific social identities.

The fourth section comprised five items from the scale developed by Monteiro et al. (2022) to assess self-esteem; for example, “I feel I do have much to be proud of.” The fifth section used three items from the scale created by Kessler et al. (2002) to evaluate psychological distress, including the item “During the last 30 days, about how often did you feel hopeless?” All four scales were rated on a five-point Likert scale ranging from 1 (Strongly Disagree, Never) to 5 (Strongly Agree, Always).

All instruments were administered in standard Mandarin Chinese. Given the linguistic and cultural diversity of the participating ethnic minority groups, the scales were not separately adapted or validated for each group’s specific cultural context. The procedures used for translation and pilot testing are described in the following subsection.

### 3.3 Instrument translation and pilot testing procedures

To ensure linguistic accuracy and conceptual equivalence, a standardized forward–backward translation procedure was employed. All questionnaire items were first translated from English into Mandarin Chinese by a bilingual researcher with a background in psychology. An independent bilingual translator, who was not involved in the initial translation, then back-translated the Mandarin version into English. The original and back-translated versions were compared, and discrepancies were discussed and resolved by the research team to ensure semantic equivalence and conceptual consistency.

Following the translation process, a pilot study was conducted with a sample of 50 adult participants drawn from the target ethnic minority populations. The purpose of the pilot test was to assess item clarity, overall comprehensibility, and the internal consistency of the translated instruments. Participants were invited to report any items they found ambiguous or difficult to understand. Based on this feedback, minor wording adjustments were made to improve clarity while preserving the original meaning of the items.

Reliability analyses based on the pilot data indicated acceptable internal consistency for all scales. Specifically, Cronbach’s alpha coefficients obtained from the pilot study were [*α* = 0.983] for perceived cultural continuity, [*α* = 0.955] for self-schemas, [α = 0.927] for self-esteem, and [α = 0.963] for psychological distress. These results supported the use of the translated instruments in the main survey.

### 3.4 Data analysis

We used AMOS 23 to specify and estimate a structural equation model (SEM) examining the associations among perceived cultural continuity, self-schemas, self-esteem, and psychological distress. Model parameters were estimated with the maximum likelihood method. Following the standard two-step procedure, we first evaluated the measurement model and then tested the structural model, assessing internal consistency, convergent and discriminant validity, global fit indices, standardized path coefficients, and indirect (mediated) effects.

To address potential common method variance (CMV) arising from self-report measures, we followed the procedure recommended by Mossholder et al. (1998) and compared two alternative models on their chi-square and degrees of freedom. Specifically, Model 1 was specified as a single-factor model in which all measurement items loaded onto one common latent factor, representing a conservative test of whether a single source of variance could account for the observed covariation among the measures. Model 2 was specified as the proposed multi-factor measurement model, in which items loaded onto their theoretically corresponding latent constructs.

Model 1 yielded a chi-square of 10172.090 with 299 degrees of freedom (*p* < 0.001), whereas Model 2 produced a chi-square of 788.082 with 269 degrees of freedom (*p* < 0.001). The large and significant difference in model fit between the single-factor model and the theoretically specified measurement model suggests that a single latent factor cannot adequately explain the covariance among the study variables. Therefore, CMV is unlikely to pose a serious threat to the validity of the study’s findings.

## 4 Results

### 4.1 Measurement model

We evaluated the measurement model using confirmatory factor analysis (CFA) in AMOS 23. As shown in Table 2, all latent constructs exhibited excellent internal consistency, with Cronbach’s α coefficients greater than 0.90, consistent with Fornell and Larcker (1981). The average variance extracted (AVE) for each construct exceeded 0.70 and composite reliability (CR) values were above 0.90, indicating strong convergent validity. Standardized factor loadings from the principal component factor analysis ranged from 0.805 to 0.953 (Table 2), further supporting construct validity. Discriminant validity was established using the Fornell–Larcker criterion: for each construct, the square root of its AVE was higher than its correlations with other constructs (Table 3).

**Table 2:** Reliability and validity.

Items	Factor loadings	Cronbach’s α	CR	AVE
Perceived cultural continuity (PCC)		0.977	0.977	0.875
PCC1	0.909			
PCC2	0.951			
PCC3	0.940			
PCC4	0.934			
PCC5	0.924			
PCC6	0.953			
Self-schemas (SS)		0.951	0.951	0.710
SS1	0.824			
SS2	0.818			
SS3	0.812			
SS4	0.823			
SS5	0.805			
SS6	0.902			
SS7	0.882			
SS8	0.871			
Self-esteem (SE)		0.958	0.958	0.820
SE1	0.885			
SE2	0.913			
SE3	0.900			
SE4	0.929			
SE5	0.901			
Psychological distress (PD)		0.963	0.963	0.814
PD1	0.859			
PD2	0.892			
PD3	0.921			
PD4	0.921			
PD5	0.885			
PD6	0.932			

**Table 3:** Pearson correlation.

Construct	PCC	SS	SE	PD
PCC	(0.935)			
SS	0.604 **	(0.843)		
SE	0.612 **	0.716 **	(0.906)	
PD	−0.484 **	−0.582 **	−0.579 **	(0.902)

The square roots of AVE are shown on the diagonal; off-diagonal elements are Pearson correlations among constructs. **p < 0.01.

### 4.2 Structural model

**Figure 2:**
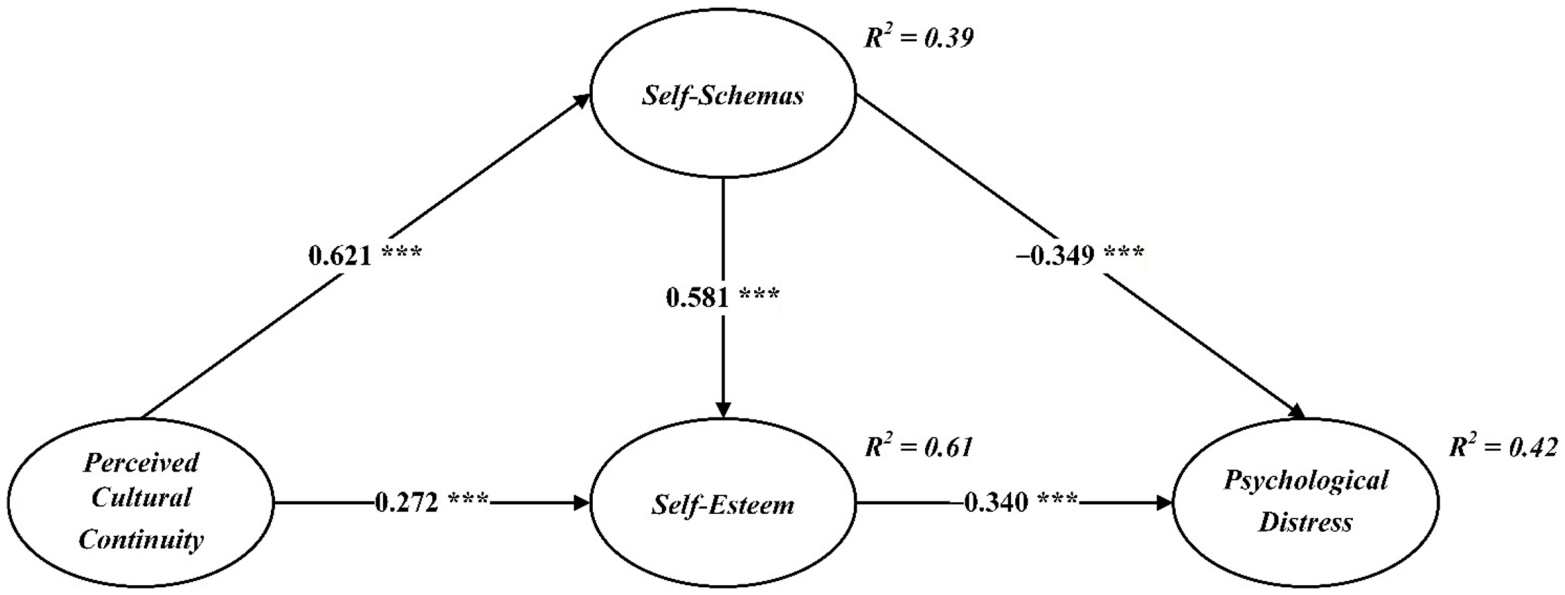
Structural model. ***p < 0.001.

After establishing the reliability and validity of the measurement model, the structural model was estimated in AMOS 23 to test the proposed hypotheses. The CFA results based on 5,000 bootstrap samples indicated good model fit (χ^2^/df = 2.944, NFI = 0.945, RFI = 0.939, IFI = 0.963, TLI = 0.959, CFI = 0.963, RMSEA = 0.065). Zero-order Pearson correlations among the study variables are reported in Table 3, and the standardized path coefficients of the structural model are shown in Figure 2.

As displayed in Figure 2, perceived cultural continuity was positively associated with self-schemas (*β* = 0.621, *p* < 0.001) and self-esteem (*β* = 0.272, *p* < 0.001), supporting H1 and H2. Self-schemas were positively associated with self-esteem (*β* = 0.581, *p* < 0.001), supporting H3, and negatively associated with psychological distress (*β* = −0.349, *p* < 0.001), supporting H4. Finally, self-esteem was negatively associated with psychological distress (*β* = −0.340, *p* < 0.001), supporting H5.

Mediation was tested using bootstrap estimation with 5,000 resamples and 95% bias-corrected confidence intervals (see Table 4). Perceived cultural continuity showed a significant indirect effect on psychological distress through self-schemas and self-esteem, with an estimated coefficient of −0.432 (SE = 0.042, 95% CI [−0.516, 0.350], *p* < 0.001), thereby supporting H6.

**Table 4:** Standardized indirect effect.

Path	Point estimate	Product of coefficients		Bootstrapping		
				Bias-corrected 95% CI		Two-tailed significance
		SE	Z	Lower	Upper	
PCC → PD	−0.432	0.042	−10.286	−0.516	−0.350	*p* < 0.001

## 5 Discussion

### 5.1 Theoretical contributions

Grounded in a structural equation modeling approach and based on data from ethnic minority adults in Southwest China whose traditional scripts are endangered, this study provides evidence of an overall pattern in which perceived cultural continuity is associated with lower psychological distress through two inter-connected self-related mechanisms: self-schemas and self-esteem. These findings position cultural continuity not merely as a contextual background condition, but as a strength-based psychological resource that may support well-being in culturally vulnerable communities.

First, this study advances research on cultural continuity and mental health by formally testing a process model that links perceived cultural continuity to psychological distress via self-schemas and self-esteem. Although prior work has highlighted cultural continuity as a protective resource (Auger, 2016; Brougham and Haar, 2013; Cobb et al., 2025), much of the evidence has relied on bivariate associations or conceptually inferred pathways. By modeling the relationships simultaneously within a latent-variable SEM framework, the present study offers stronger evidence that cultural continuity is closely linked to internal psychological resources, rather than being related only to general attitudinal orientations (Sani et al., 2007; Sani et al., 2008).

Second, this research refines self-continuity theory by specifying its internal cognitive and evaluative mechanisms at the collective level. Self-continuity theory emphasizes temporal coherence for psychological well-being (Sedikides et al., 2023), yet empirical work has often treated continuity as a global perception without clearly identifying how it becomes embedded in the self-system. By demonstrating that perceived cultural continuity is positively associated with both self-schemas and self-esteem, and that these constructs statistically account for its relationship with psychological distress, the present study clarifies how collective continuity is reflected in individual psychological stability. This refinement extends self-continuity theory beyond personal life narratives and suggests that continuity derived from endangered cultural traditions can be cognitively organized and emotionally evaluated through stable self-structures (Ji et al., 2022; Cruwys et al., 2021).

Third, this study extends social identity theory by highlighting the importance of temporal continuity as a distinct identity-relevant resource that contributes to well-being. Social identity theory has typically focused on the strength and positivity of group identification (Brown, 2000; Hogg, 2016; Turner and Oakes, 1986; Stets and Burke, 2000), often without explicitly considering the perceived durability of group traditions and historical narratives. By modeling perceived cultural continuity as a latent construct and linking it to self-schemas, self-esteem, and psychological distress, this study demonstrates that the temporal stability of cultural identity constitutes a distinct psychological resource. The findings suggest that social identification is not only about belonging in the present but also about perceiving one’ s cultural group as enduring over time (Amiot et al., 2015; Firestone et al., 2024). This extension broadens the theoretical scope of social identity theory and highlights continuity as an important, yet previously underexamined, dimension of identity-based mental health processes.

Taken together, this study contributes to identity and mental health research by offering an empirically tested, theory-driven model that integrates collective cultural continuity with individual psychological outcomes through a pattern of associations among self-related mechanisms. Methodologically, the use of structural equation modeling allows for a rigorous examination of simultaneous direct and indirect effects, strengthening theoretical claims about how culture, identity, and mental health are interconnected. Substantively, the findings position perceived cultural continuity not merely as a contextual or background factor, but as an active psychological resource shaping self-structure and emotional adjustment, particularly in culturally vulnerable populations. It should be noted that these conclusions are based on overall associations observed in the pooled sample, and potential variations across ethnic or demographic subgroups were not examined in the present study.

### 5.2 Practical implications

This study demonstrates that perceived cultural continuity, coherent self-schemas, and higher self-esteem constitute important psychological resources for ethnic minorities whose traditional scripts and languages are endangered. The findings have several practical implications for policymakers, educators, community organizations, and mental health practitioners working with minority populations in Southwest China.

First, at the cultural and policy level, protecting and revitalizing endangered scripts and associated cultural practices can serve as both heritage preservation and mental health promotion. For example, community-based initiatives such as the “Dongba Script Cultural Transmission Project” in Lijiang engage local youth and elders in learning and practicing the Naxi Dongba script, while Yi language curricula in Liangshan Prefecture incorporate regional dialects and writing systems into formal education. Programs like these enhance visibility and everyday engagement with minority scripts, reinforcing perceptions of cultural continuity, which this study found to be linked to more coherent self-schemas, higher self-esteem, and lower psychological distress. Policy measures may include expanding bilingual education, supporting public signage in minority scripts, promoting documentation and digitalization of writing systems, and facilitating access to cultural resources in schools and communities. These strategies help ensure that scripts and languages remain actively used and culturally valued across generations.

Second, the mediating roles of self-schemas and self-esteem indicate that cultural revitalization efforts should be explicitly linked to identity-building and empowerment. Community programs such as intergenerational storytelling, life-narrative workshops, cultural festivals, and youth leadership initiatives rooted in local traditions provide opportunities for participants to integrate collective history and cultural knowledge into personal identity. Partnerships with established institutions, such as the Yunnan Provincial Ethnic Affairs Commission’s Minority Language Guidance Committee, and grassroots initiatives led by local cultural practitioners and artists, can enhance program reach, legitimacy, and sustainability. By engaging in these activities, individuals can reflect on their ethnic identity, script, and heritage, thereby reinforcing psychological resources that buffer against distress. Schools serving minority students can complement these efforts by implementing culturally responsive curricula that validate minority scripts and histories, rather than marginalizing them.

Third, mental health services in minority regions should incorporate cultural sensitivity and structural awareness. Counselors and social workers can integrate discussions of cultural continuity, identity, and pride into interventions, helping clients reframe experiences of marginalization or script endangerment in ways that promote resilience. Recruiting and training mental health professionals from minority backgrounds and providing services in local languages can further strengthen trust and intervention effectiveness. These approaches enable psychological support to be closely aligned with cultural preservation, creating a synergistic effect between mental health promotion and cultural continuity.

Finally, these findings highlight the importance of cross-sector collaboration. Efforts to reduce psychological distress cannot be separated from broader initiatives addressing educational inclusion, equitable resource allocation, and sustainable cultural development. By coordinating strategies across cultural preservation departments, educational authorities, health systems, and local community organizations, interventions can simultaneously support minority language use, reinforce identity and self-concept, and enhance well-being. Connecting these local and regional initiatives to broader frameworks of inclusive development, quality education, and public health strengthens their translational impact and situates the research within a global discourse on minority rights and psychological well-being.

### 5.3 Limitations

Despite its contributions, this study has several limitations that should be acknowledged. First, the use of a cross-sectional design limits causal inference regarding the relationships among perceived cultural continuity, self-schemas, self-esteem, and psychological distress. Although the proposed structural equation model is theoretically grounded, the directionality of the associations cannot be confirmed. Future longitudinal or experimental studies are needed to examine temporal and causal dynamics.

Second, all variables were assessed using self-report questionnaires, which may be subject to common method variance and social desirability bias. Despite applying established procedures to mitigate common method bias, residual effects cannot be ruled out. Given the politically sensitive nature of ethnic and cultural issues in the research context, some participants may have underreported psychological distress or cultural dissatisfaction, potentially attenuating the observed associations.

Third, the reliance on convenience and snowball sampling constrains the representativeness and generalizability of the findings. These approaches may have led to the overrepresentation of socially connected or culturally engaged individuals, limiting the extent to which the results can be generalized across the Tibetan, Yi, Dai, and Naxi populations or to other minority contexts. Future research should consider probability-based sampling or mixed-method designs to enhance external validity.

Fourth, although a forward–backward translation procedure and pilot testing were conducted, the measurement instruments were not specifically adapted or independently validated for the distinct cultural and linguistic contexts of the four ethnic groups. As all measures were administered in standard Mandarin Chinese, subtle cultural or linguistic nuances may not have been fully captured. Accordingly, the findings should be interpreted with caution, particularly in cross-group comparisons.

Finally, the exclusive reliance on quantitative survey data may limit the depth of contextual understanding. Future research could adopt a mixed-methods approach by incorporating in-depth qualitative interviews to complement and contextualize the quantitative findings, thereby enhancing interpretive richness and validity.

## 6 Conclusion

This study shows that, among ethnic minorities with endangered scripts in Southwest China, higher perceived cultural continuity is associated with more coherent self-schemas, higher self-esteem, and lower psychological distress. Self-schemas and self-esteem jointly emerged as key psychological pathways linking cultural continuity to mental health. These findings extend existing research on cultural continuity by focusing on minorities facing the marginalization of their own languages and scripts, and suggest that preserving and revitalizing cultural traditions may have not only cultural and historical value but also important psychological benefits. At the same time, the cross-sectional and self-report nature of the data means that causal inferences should be drawn with caution, and future longitudinal and mixed-methods studies are needed to deepen understanding of how cultural continuity can be translated into sustainable mental health support for minority communities.

## Data Availability

The raw data supporting the conclusions of this article will be made available by the authors, without undue reservation.
