# Correction: Perceived cultural continuity, self-schemas, self-esteem, and psychological distress among members of ethnic groups with endangered scripts in Southwest China

**DOI:** 10.3389/fpsyg.2026.1827131

**Published:** 2026-03-23

**Authors:** Xian Zhang, Zichan Li

**Affiliations:** 1 Academic Affairs Office, Hunan First Normal University, Changsha, China; 2 Department of Chinese Music, Singapore Raffles Music College, Singapore, Singapore

**Keywords:** southwestern ethnic minorities, perceived cultural continuity, psychological distress, self-esteem, schema construction

In the published article, an incorrect grant number was provided for National Social Science Fund of China. The correct number is 23BMZ151.

The original version of this article has been updated.

